# Risk of Cardiovascular Disease in Male Farmers Over the Age of 45: A Review of Literature

**DOI:** 10.7759/cureus.24642

**Published:** 2022-05-01

**Authors:** Binoy Desai, Sidharth Sahni, Harrison Jordan, Raghav Sahni, Ryan Reinbeau, Alan Nguyen, Olumide Babalola

**Affiliations:** 1 Internal Medicine, Rowan University School of Osteopathic Medicine, Stratford, USA; 2 Emergency Medicine, Crozer-Chester Medical Center, Upland, USA; 3 Internal Medicine, West Virginia School of Osteopathic Medicine, Wheeling, USA; 4 Interventional Spine and Sports Medicine, Cantor Spine Center at the Paley Orthopedic and Spine Institute, Fort Lauderdale, USA; 5 Physical Medicine and Rehabilitation, Larkin Community Hospital, South Miami, USA; 6 Physical Therapy, University of the Sciences, Philadelphia, USA

**Keywords:** awareness of cardiovascular disease, cardiovascular risk (cvr), traditional cardiovascular risk factors, occupation health, farmer, cad: coronary artery disease, cardiovascular prevention, myocardial infarction, rural area, cardiovascular risk factor

## Abstract

The prevalence of heart disease in farmers is well documented, but there is limited research characterizing the diverse risk factors associated specifically with male farmers over the age of 45 in the United States, while also providing a multifactorial strategy to address these concerns. The majority of current research either focuses on the general rural population or does not take into account different demographic variables. Hence, this review looked to address those gaps by focusing on those specific variables.

A literature review was generated looking at risk factors associated with cardiovascular disease in farmers using key search terms. Next, an additional search was conducted focusing on treatment plans to address these concerns. The articles were then sorted based on the inclusion and exclusion criteria. The initial articles were sorted by one researcher and then reassessed on two separate occasions. The literature review was performed using these databases: PubMed, CINAHL, Cochrane, and Ovid Medline. A total of 221 articles were generated, of which 12 articles matched the criteria.

The articles highlighted important risk factors that were either social or non-social determinants of health that negatively impacted the target population. These were followed up by offering solutions that attempted to provide a holistic approach, including clinical and community-based interventions.

Male farmers over the age of 45 years are at an increased risk of being diagnosed with heart disease compared to non-farmers in the same demographic. When attempting to implement interventions, stress management should be incorporated into the treatment plan. In addition, a multifaceted approach targeting clinical and community concerns is recommended.

## Introduction and background

Heart disease is one of the leading causes of death in rural male farmers. Heart disease is an umbrella term used to describe a variety of diseases that affect the heart [1,2]. Coronary artery disease (CAD), heart failure with preserved ejection fraction (HFpEF), heart failure with reduced ejection fraction (HFrEF), and arrhythmias all fall into the category of heart disease [3]. Moreover, a heart attack, also referred to as a myocardial infarction (MI), occurs when ischemia results in the death of myocardial tissue [4]. MI can be a direct result of heart disease, and 80-90% of MIs are caused by a coronary artery thrombus in an area where previous damage has occurred [4]. There are numerous modifiable and non-modifiable risk factors associated with heart disease, and these risk factors can help predicate the likelihood of having long-term complications associated with heart disease [3,4]. Certain populations, such as farmers, are more prone to heart disease when compared to their counterparts [2,3].

As per the 2017 Census of Agriculture conducted by the United States Department of Agriculture, the number of farming producers in 2017 has increased by 7% since 2012 [5]. The average age of a farmer was listed at 57.5 years old, demonstrating an increase of 1.2 years since 2012, with a majority being between the ages of 45 and 64. Furthermore, 64% of farmers were found to be men. These statistics demonstrate an upward trend in the prevalence of male farmers over the age of 45. With an increase in this target population, there seems to be a need to address the growing concern of the higher-than-normal prevalence of heart disease [5,6]. Though there is available literature recognizing the prevalence of heart disease in farmers, there is limited evidence that identifies preventable risk factors while offering practical long-term solutions that address heart disease holistically for middle-aged male farmers over the age of 45. Thus, our aim is to review the current literature regarding the elevated risk of cardiovascular disease in this population and to explore how to potentially reduce this risk.

## Review

Methodology

The purpose of this study was to review literature pertaining to heart disease in rural male farmers over the age of 45. We wanted to see what specifically caused them to be at an increased risk of heart disease and if there were ways to possibly improve health outcomes.

The databases used for this study were: PubMed, CINAHL, Cochrane, and Ovid Medline. The search terms used to find current studies were: heart disease, farmer, male, farming, risk, stress, screening, treatment, therapy, intervention, language, lifestyle, health wellness, education, financial debt, stressors, age, work-related stress, CAD, MI, arrhythmias, heart infections, congenital heart defects, health belief model, clinical interventions, behavioral therapy, screening, stress reduction, nutrition, smoking, policy, and social change. The search was conducted first to look for risk factors and then for treatment options.

There were clear inclusion and exclusion criteria to better organize this study. Only studies conducted in the United States were included in the review. The inclusion criteria specified that articles meeting the following requirement be included: heart disease in male farmers over the age of 45. After meeting the initial criteria, articles that focused on trends, risk factors, prevention, pathologies, and treatments related to the specific population were included. Selection criteria specifically excluded articles that failed to include information that was applicable to the target population or showed a change in heart condition due to organic, pesticidal, or other external toxic factors.

**Figure 1:**
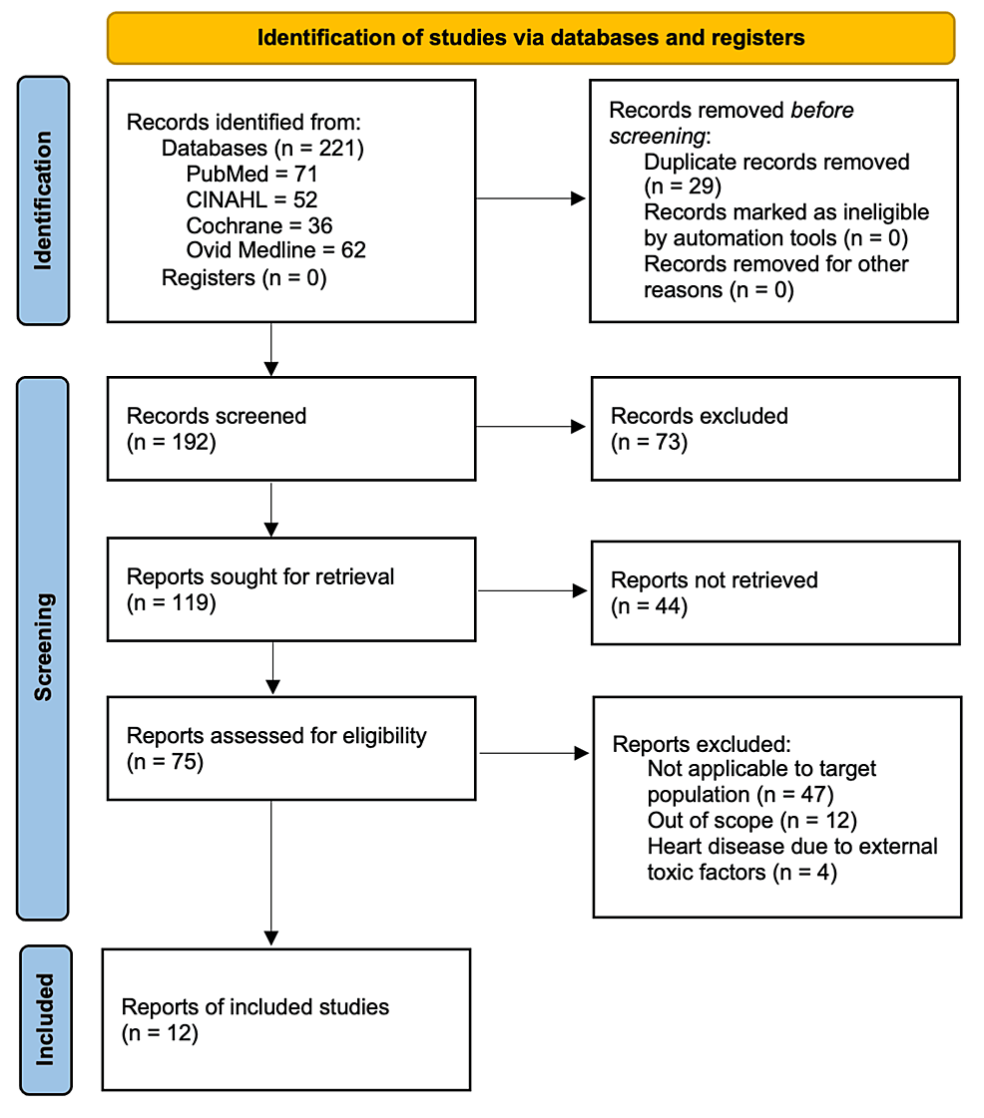
PRISMA flow diagram PRISMA: Preferred Reporting Items for Systematic Reviews and Meta-Analyses

The studies included in this literature review consisted of peer-reviewed studies, primary research, empirical studies, systematic reviews, and research on critically appraised topics. Our search located 221 articles, of which 75 were analyzed, resulting in 12 articles that met the criteria for inclusion in our study (Figure 1). The initial articles were sorted by one graduate student researcher and then reassessed by him on two separate occasions. Sample sizes varied in each of the studies reviewed by the researcher, which is important to consider.

Results

After investigating the articles, it was found that specific social and non-social determinants predicted risk factors and trends associated with cardiovascular disease in middle-aged farmers. Medical research suggests that although farmers may be healthier than the general population, they experience high rates of cardiovascular disease and cancer [2]. Heart disease is the number one cause of death in our target population of rural, male farmers in the United States [1]. The Agriculture Health Study’s (AHS) website investigates the causes of death of private farmers [1]. The AHS specifically looks at farmers from Indiana and North Carolina. 

Increased age is another well-documented risk factor related to the increased incidence of heart attacks in this population. Men over the age of 45 and women over the age of 55 are at an increased risk of having a heart attack [7]. In addition, individuals over the age of 65 (19.8%) have the greatest prevalence of heart disease, followed by those individuals between the ages of 45 and 64 years (7.1%), and lastly by those between the ages of 18 and 44 years (1.2%) [8,9].

According to the Centers for Disease Control and Prevention (CDC), although the prevalence of heart disease is decreasing, it still accounts for one in five deaths [10,11]. The decrease in prevalence hopefully does not conceal the continued disparities in disease based on demographic characteristics and geography [11]. The prevalence has shifted and varies substantially based on sex, race, education, and state of residency. It was noted that men with lower education residing in southern states were overall at a higher risk than before [10,11]. Thus, programs should focus more on specific states and populations with increased prevalence, such as male farmers over the age of 45 living in rural settings.

From national statistics, it is clear that heart disease is more prevalent in rural areas as compared with metropolitan ones [12]. The traditional vision of Midwestern and Southern farmers is of fit, physically active men whose daily activities involve manual labor, although conventional farming models and activities have been adapted. A modern-day farmer uses robotic technology instead of physical labor to cover their fields with fertilizer, pesticides, and water. Drones and global positioning systems replace the act of physically walking to survey the field and monitor climate conditions [12].

Chronic uncontrolled hypertension (high blood pressure) is the most common cause of HFpEF or diastolic heart failure. Two common underlying causes of HFrEF, or systolic heart failure, are uncontrolled hypertension and coronary artery disease [2]. Therefore, for farmers to avoid being at risk of heart failure, they need to improve their cardiovascular health. The American Heart Association defines cardiovascular health as the presence of ideal health behaviors and characteristics such as physical activity, healthy diet, nonsmoking, normal body mass index, and normal cholesterol, blood glucose, and blood pressure levels [12]. There are no studies in the current literature that discuss the prevalence of heart failure, either systolic, diastolic, or combined, among male farmers in the United States. Future research addressing cardiovascular health in rural environments could expand on such a topic as it relates to heart failure. However, the United States national data examining cardiovascular disease and heart failure risk behaviors across occupational categories reported that the category which included farmers had the highest prevalence of poor scores for diet (84.3%) along with a moderate prevalence of poor scores for the weight (68.7%) and physical activity (47%) [13].

Additionally, a qualitative study found that rural men's view of physical activity is that it is part of their daily routine and that structured exercise sessions are not practical or feasible [14]. Focus groups of men in rural communities found that they prefer meat-based meals, which are associated with hunting and fishing as part of their lifestyle [14]. Some evidence exists that interventions to decrease sedentary behavior, improve diet, and promote exercise will benefit the health of those in rural communities, but further exploration to discover how these measures reduce the prevalence of heart failure in this population could be valuable [15,16].

Heart disease is associated with modifiable and non-modifiable risk factors, as stated earlier in this paper. These risk factors can also be characterized as social or non-social determinants of health. Common social determinants of health related to the target population's having an increased risk of heart disease include the occupation status of being a farmer, having limited healthcare access, education level, access to healthcare, gender, health literacy, and potential language barriers [6]. The non-social determinants of health include age, comorbidities, lifestyle, behavioral perception of heart disease, and stress. Studies have shown that the risk of heart disease from elevated stress increases with age [17-19]. Even though most farmers understand the severity of having a MI, they believe they are not susceptible to MI because of their perceived healthy lifestyles [20].

Social determinants of heart disease

Farming is considered to be one of the most dangerous jobs in the country due to the many risk factors associated with the occupation [1]. The goal should be to work with rural male farmworkers to inform them of the risks of heart disease. Although several cardiovascular health concerns exist with farming, the majority can be prevented by reducing these risk factors and conducting early screening for farmworkers [1]. 

Farmers spend much of their time operating heavy machinery as well as lifting and carrying heavy items as part of their daily activities. These factors, including the constant pressure to work longer and harder, contribute to increased stress levels and tobacco abuse, which are major risk factors for heart disease [21,22]. Furthermore, unpredictable and frequent changes in seasons, unexpected plant and animal diseases, and financial issues, including debt, and downturns in the economy, are also listed as primary stressors for farmers [22]. Rural farmers exposed to these risk factors may have to travel long distances to receive healthcare, which may deter farmers from scheduling frequent visits to healthcare practitioners.

Therefore, tailoring wellness and prevention programs for rural farmers is crucial to their health and longevity. Making sure that programs are customized for their lifestyle and reading proficiency will improve adherence. The National Center for Farm Workers’ Health (NCFH) reports that about 35% of rural farmers indicate not being able to speak English at all, highlighting the need for low-literacy level handouts available in different languages [23]. Having interpreters available during presentations, providing charts, and distributing illustrations that are easy to follow will also help in delivering quality care to these individuals.

In addition, encouraging rural farmers who currently have no health insurance to enroll in one would greatly impact their health outcomes. Thirty-six percent of rural farm workers reported being covered by unemployment insurance. Fifty-four percent claimed they were not covered, and 8% did not know if they were covered [23]. Even though educating the farming population should be the primary goal of preventing heart disease, it is important for them to understand the importance of health insurance and how they can get coverage. These are only a few examples of risk factors that have the potential to be addressed to improve the health of rural farmers.

Non-social determinants of heart disease

Heart disease is preventable; therefore, in an effort to reduce the risk of heart disease, many health promotion programs have been created [24]. The leading modifiable risk factors include high blood pressure, high cholesterol, poor diet/physical inactivity, cigarette smoking, obesity, and diabetes. These risk factors damage coronary and blood vessels over time, so it is important to address them early to prevent potentially devastating complications [24]. Diet and physical activity, control of high blood pressure and cholesterol, smoking cessation, and appropriate aspirin use are key factors in reducing the risk of heart disease, especially heart attacks [24]. 

The leading risk factor for heart attacks in rural, male farmers is stress, specifically financial stress [9,10]. When a person is under a great deal of stress, their blood pressure and cholesterol can rise. In addition, they are less likely to engage in physical activity [17,25]. Chronic stress can trigger elevated levels of stress hormones like adrenaline and cortisol which increase one’s risk of cardiovascular complications. Studies also link stress to changes in the way blood clots, which increases the risk of heart attacks [25]. The wellness program listed here will focus on stress relief for patients, as well as the incorporation of physical activity.

Clinical Interventions 

Some of the current health promotion interventions for heart disease and improving atherosclerotic cardiovascular disease (ASCVD) risk are listed below [24]. The focus was on programming that could relate to the specific adult population, though it should be noted that there are many more programs that exist for the reduction of heart disease in other demographics (e.g., Jump Rope for Heart is a health promotion program aimed at cardiovascular fitness for children) [24,26]. The highlighted programs are ones that can be incorporated into an eight-week program.

Clinical Recommendations

Aspirin Use for the Prevention of Cardiovascular Disease

In an April 2022 statement, the United States Preventive Services Task Force (USPSTF) recommends at a grade C level that the decision to initiate low-dose aspirin use for the primary prevention of cardiovascular disease in adults between the ages of 40 and 59 years who have a 10% or greater 10-year ASCVD risk should be made individually [27]. The USPSTF reports that evidence suggests that the net benefit of aspirin use is small in this population. The USPSTF also reports that individuals who are not at increased risk for bleeding complications and are willing to take low-dose aspirin daily are more likely to benefit [27]. The challenge here will be getting patients to comply and remember to take their aspirin. At a grade D level, the USPSTF recommends against initiating low-dose aspirin use for the primary prevention of cardiovascular disease in adults 60 years of age or older [27].

Statin Use for the Prevention of Cardiovascular Disease

At a grade B level, the USPSTF recommends that adults without a history of cardiovascular disease use a low-to-moderate dose statin for the prevention of cardiovascular disease events and mortality when these three criteria are met: age between 40 and 75 years, one or more cardiovascular disease risk factors (e.g., dyslipidemia, hypertension, diabetes, or smoking), and a calculated 10-year ASCVD risk of 10% or greater [28]. At a grade C recommendation level, if the same criteria are met for an individual in the same age group but the calculated 10-year ASCVD risk is between 7.5% and 10%, the likelihood of benefit is smaller and it is up to the clinician whether to offer the low-to-moderate dose statin or not [28].

Behavioral Counseling in Primary Care to Promote a Healthy Diet and Physical Activity

At the grade B level, the USPSTF recommends offering or referring behavioral counseling interventions to adult patients with risk factors for cardiovascular disease to promote physical activity and a healthy diet [29]. The concern with this is that many farmers may be resistant to going to the doctor’s office until something is seriously wrong with them; establishing care first and then offering house visits would be beneficial if it were possible.

Know the ABCs of Heart Attack Prevention

The ABCs of heart attack prevention are simple lifestyle changes that one can make to reduce their risk and live a longer, stronger life [26]: A - avoid tobacco, B - become more active, and C - choose good nutrition [26].

Screening for High Blood Pressure in Adults

Adults 18 years of age and older should be screened for high blood pressure during yearly checkups since it is the number one modifiable risk factor for CAD [6,18].

Screening for Lipid Disorders in Adults

At a grade A level, the USPSTF strongly recommends routinely screening men ages 35 and older for lipid disorders and treating abnormal lipids in those who are at an increased risk of coronary heart disease [30]. At a grade B level, the USPSTF recommends routinely screening men between the ages of 20 and 35 for lipid disorders if they have other risk factors for coronary heart disease (e.g., diabetes, family history of cardiovascular disease in male relatives before the age of 50 and in female relatives before the age of 60, family history suggestive of familial hyperlipidemia, or multiple coronary heart disease risk factors such as tobacco use and hypertension) [30].

Screening for Prediabetes and Type 2 Diabetes in Adults

At a grade B level, the USPSTF recommends screening for prediabetes and type 2 diabetes in adults between the ages of 35 and 70 who are overweight or obese [31]. The American Diabetes Association recommends the fasting plasma glucose test or hemoglobin A1C level for screening [31]. Identifying and treating diabetes will undoubtedly decrease the male farmer's ASCVD risk.

Community-based intervention

Behavioral and Social Approaches to Increase Physical Activity

Individually-adapted health behavior change programs: A physical therapist, personal trainer, or healthcare professional can put together an individualized fitness program that focuses on reducing the risks of heart disease [32,33]. This is a great program because it gives people the knowledge and tools needed to improve their health [24].

Social support interventions in community settings: This kind of social support can keep a person motivated with their risk reduction program, offer new knowledge/education, and is a good place to voice any opinions, concerns, or questions [24].

Campaigns and Informational Approaches to Increase Physical Activity

Community-wide campaigns: This is a valuable way to raise awareness of heart disease. People can learn what it is, who is at risk, and how it can be prevented [24]. The community can work together to decrease each person’s risk and provide support for one another [24].

Health Communication and Social Marketing

Health communication campaigns including mass media and health-related product distribution: Mass media is a great way to inform a large number of people at once about heart disease and how it can be prevented [17]. Product distribution is beneficial because it eliminates the excuse that someone does not have the equipment or knowledge to prevent heart disease [17]. Product distribution can range from flyers and brochures (education) to pedometers (measure physical activity) and stress relief balls (reduce stress and in turn reduce some risk factors for heart disease) [24,32].

Online support/tools: Different websites, such as the American Heart Association, offer tools to help people reduce their risk of heart disease. For example, there are calculators online that determine the risk of a heart attack. There are also heart-healthy quizzes to improve one’s know-how for fighting off heart disease. Additionally, one could use the My Life Check health assessment, which will help draft a personalized strategy for leading a healthier life [26]. These online tools are important in today’s society because most people get their information from the computer. However, online tools are not helpful for people who do not have computers or the internet, thus risking an increase in health disparities [26].

Obesity Prevention and Control

Worksite programs: Decreasing obesity will greatly decrease one's risk not only of heart disease but also of many other serious health conditions [11]. Focusing on worksite programs is important because many people think they do not have the time to participate in risk reduction programs, but incorporating it into their workday will give them the opportunity and knowledge to start [34].

Worksite Health Promotion

Assessment of health risks with feedback to change employees’ health: This helps to give more people the knowledge/education they need to decrease their risk of heart disease [24,34]. This also eliminates the pitfall of people not knowing whom to go to for help and information. Sometimes incentives are even given to employees who participate in risk reduction programs [24].

Stress reduction

Stress reduction does not get as much attention in health promotion programming as physical activity does for reducing one’s risk of heart attacks; this is partly because exercise helps to reduce stress [25,33]. Some examples of stress coping skills are exercise, relaxation, proper nutrition, therapy, self-responsibility, and setting realistic goals. Healthcare professionals can provide a wide range of activities that focus on the development of any of the above coping skills [33]. Stress reduction programs are going to be very important for our population.

Program strategies

The American Heart Association does a great deal to advocate for heart health [26]. Some of the other programmings they continue to work on include smoke-free public places, better nutrition and high-quality physical education in school, more walkable and bikeable streets, roads, and parks, as well as affordable and available healthcare for all [22-26].

There is an abundance of health promotion programming focusing on the reduction of risk factors for heart disease and heart attacks [4,19]. New programming should focus more on social and economic inequalities in health [17,20]. Health disparities are a serious barrier to achieving the goals of health promotion programming [34,35]. The table below explains why farmers do not participate in the healthy behaviors needed to decrease their risk of MI and how healthcare professionals can address the concerns (Table 1).

**Table 1:** Health belief model to reduce the risk of myocardial infarction in male farmers MI: myocardial infarction

Concept	Definition	Program Strategies
Perceived susceptibility	Beliefs about the chances of getting an MI: The Agricultural Health Study (a long-term study of Iowa and North Carolina farmers) found that 1,789/52,394 private farmers and 122/4,916 commercial farmers have experienced a MI [24]. Because of their generally healthy lifestyles, farmers believe they are not susceptible to MIs [4].	We are going to assess the patient's risk of getting a MI by looking at the patient's controllable risk factors: smoking, high blood pressure, high cholesterol, diabetes, being overweight or obese, physical inactivity, and self-reported stress [24]. The Heart Attack Risk Calculator can be used, as well as questionnaires assessing a patient's stress level [22,25].
Perceived severity	Beliefs about the seriousness of MI and its consequences: Most farmers understand the severity of MI, but they do not believe it will happen to them [4,20].	A major consequence of a MI is death. The use of statistics as a scare tactic can help us demonstrate the severity and seriousness of a heart attack: each year over a million people in the United States have a heart attack, and about half of them die [24].
Perceived benefits	Beliefs about the effectiveness of taking action to reduce risk: Farmers can be skeptical about stress reduction techniques and their ability to decrease risk of MI [17,35].	A stress reduction and physical activity program will be implemented to reduce controllable risk factors (decrease blood pressure, cholesterol, and weight), decreasing the risk of a MI [17,25]. By using data from previous studies, we will help farmers to see the perceived benefits of our programming.
Perceived barriers	Beliefs about the material and psychological costs of taking action: Many farmers believe they will not have time in the day to incorporate these strategies [4,20].	We will work with the patient to figure out what the patient's perceived barriers are and brainstorm ways to overcome them. This could include performing exercises in little increments throughout the day if short on time, or performing stress reduction strategies before bed if unable to find a quiet area during the day [17,25].
Cues to action	Factors that activate “readiness to change” [4]	Wellness materials will be given on stress reduction and physical activity. We can also set reminders on the patient's phone to help with compliance [17].
Self-efficacy	Confidence in one’s ability to take action[4]	Training and guidance will be given throughout the program, realistic goals will be set, progress will be charted to increase motivation, and verbal reinforcement will be given [17]. To increase confidence in one’s ability to take action beyond our program, we will implement a maintenance program and provide periodic follow-up.

Discussion

This study focuses on social and non-social factors that disproportionately affect the target population of rural male farmers over the age of 45. The study highlights important concerns and offers interventions that address both clinical and community-based concerns. In addition, it emphasizes the importance of stress management, which is a common risk factor that might get overlooked at times.

Interestingly, a study by Prokosch et al. found that the median income and the percentage of the particular population in poverty explained 50% of the variation in adjusted premature mortality rates [3]. Controlling for these socioeconomic variables largely eliminated the disparities discovered in rural populations. This study did not meet the inclusion criteria. However, the study could be valuable for future research.

The American Heart Association recommends that heart attack prevention begins by age 20 [26]. By age 40, it is even more important to incorporate some sort of risk prevention program into one's daily life. Many first-ever heart attacks are fatal or disabling so preventive measures should be taken as early as possible [26]. Since the population being addressed here is above the age of 45, it is important for a risk prevention program to begin immediately if not initiated already. Therefore, this research can be utilized by those looking to make an impact on such a population. From a small-scale rural physician striving to address health concerns in older male farmers to public health professionals or law and policymakers interested in enacting large-scale changes, this literature review will undoubtedly be valuable to accomplishing either. In addition, it can help set a baseline for future research that can focus on rural health disparities as a whole, realizing the importance of including socioeconomic variables in analyses.

Limitations

In developing this review, there were no ethical concerns. However, because this study reviewed other articles, compiled their results, and presented them in a single-source manner, there may be some human bias. One obstacle that was faced in researching this topic was the limited amount of data pertaining to it. The total number of results that fit the inclusion criteria was limited. There was also limited research on the impact of large-scale policy changes on improving cardiovascular status among populations.

## Conclusions

Heart disease is a term that describes a wide variety of diseases associated with the heart. Male farmers over the age of 45 have a higher risk of age-adjusted mortality and disability related to heart disease. The stress levels farmers endure have negative impacts. These stress levels stem from financial difficulties (unreliable prices, crop loss, debt, etc.), changes in weather, machinery breakdown, and conflicts within the farming industry itself. The results of this literature review demonstrate the clear benefits of integrating stress management when creating a treatment plan.

In addition, both clinical and community-based interventions that utilize a health belief model should be employed to make long-term changes. Steps need to be taken to change the current perceptions of cardiovascular health in the community. Male farmers tend to ignore their susceptibility to heart disease. They disregard the severity of heart disease and its fatal complications. Living in rural settings, lacking healthcare literacy, and overall disinterest in the topic are just a few ways ideal healthcare outreach and treatment is limited in this demographic. The program strategies proposed in this review provide concrete ways to alter these perceptions and mitigate risk. The combination of clinical changes with community-level interventions could lead to optimal cardiovascular outcomes in adult male farmers.
